# An all-optical soliton FFT computational arrangement in the 3NLSE-domain

**DOI:** 10.1007/s11047-017-9642-1

**Published:** 2017-10-04

**Authors:** Anastasios G. Bakaoukas

**Affiliations:** Computing and Immersive Technologies Department, University of Northampton, St. Georges Avenue, Northampton, NN2 6JB UK

**Keywords:** Solitons, 3NLSE domain, All-optical FFT, Cubic non-linear Schrödinger equation, Soliton collisions, Soliton computational schemes

## Abstract

In this paper an all-optical soliton method for calculating the Fast Fourier Transform (FFT) algorithm is presented. The method comes as an extension of the calculation methods (soliton gates) as they become possible in the cubic non-linear Schrödinger equation (3NLSE) domain, and provides a further proof of the computational abilities of the scheme. The method involves collisions entirely between first order solitons in optical fibers whose propagation evolution is described by the 3NLSE. The main building block of the arrangement is the half-adder processor. Expanding around the half-adder processor, the “butterfly” calculation process is demonstrated using first order solitons, leading eventually to the realisation of an equivalent to a full Radix-2 FFT calculation algorithm.

## Introduction

Computational systems based on soliton collisions for transferring and processing data continues to be a topic which stands at the forefront of scientific research (Jakubowski et al. 1996, 1997, 2001; Bakaoukas and Edwards 2009b; Steiglitz 2000).

Within this framework, an earlier version of this paper originally appeared in Bakaoukas (2016). The current paper has augmented this original work by including an extensive discussion of the optical solitons background theory as presented in the remainder of this section, a full explanation of all the basic concepts and parameters involved in the formulation of the computational system proposed for the 3NLSE-domain (Sect. 2), and a detailed analysis of all the numerical methods currently available for the simulation of optical solitons propagation down an optical fibre when the propagation parameters applicable to the system are those of the 3NLSE-domain (Sect. 3). The half-adder processor scheme proposed and discussed in the remainder of the paper, as well as individual soliton arrangements in the overall computational system, have been extensively tested and successfully verified using all these numerical methods.

Generally there are two different types of solitons, delineating the areas of general interest to create computational systems from. The, so called, “spatial solitons” and the “temporal solitons”, respectively defining the “spatial solitons computational systems” and the “temporal solitons computational systems”. Solitons owe their existence to the physical alignment between a phenomenon known as “Kerr non-linearity” (self-phase modulation) and the phenomenon of “chromatic dispersion” in optical fibres, which is the primary material allowing for solitons generation and propagation.

Temporal solitons in optical fibres can be described very accurately by the “cubic non-linear Schrödinger equation” or “3NLS equation” for sort, while spatial solitons can be described very accurately by the “general NLS equation” which, although describing a system which is not generally integrable, never the less enables us to calculate more accurately more complex phenomena. Solitons in both the temporal and the spatial domain can either “interact” with each other (soliton interactions) or “collide” with each other (soliton collisions).

Soliton systems which are using fast digital logic gates and carry and process information through soliton collisions have been proposed for some time now (Jakubowski et al. 1996, 1997, 2001; Bakaoukas and Edwards 2009a; Steiglitz 2000; Blair 1998). The logic gates arrangements used by these systems take advantage of the phase difference or the frequency difference emerging after each collision between orthogonally polarised solitons to represent a bit as we know it from classical computational theory. Other systems of logic gates arrangements have also been proposed, which make use of the time difference (“soliton trapping”) or of the position difference (“soliton dragging”) emerging between the solitons involved after they have clash. Particle machines, which perform calculations using soliton collisions, have been also proposed (Steiglitz 2000). The goal here is to create a global computational system, which will generally use logic gates of the type initially introduced by Toffoli (1980) or alternatively of the type initially introduced by Fredkin and Toffoli (1981) for processing and storing data very close to the classical.

Most of the major studies been carried out so far in the direction of computing with solitons are mostly at a purely theoretical level, especially considering collisions between first order solitons. Independent and more or less equally effective numerical methods like: the “Finite Difference Runge–Kutta Technique” (FDRKT), the “Split-Step Fourier Transform” (SSFT), the “Fourier Series Analysis Technique” (FSAT), and the “Fuzzy Mesh Analysis Technique” (FMAT) have been successfully applied to provide simulations of solitons propagation down the optical fibre in the 3NLS equation domain and in general have been extensively used in theoretical research on colliding solitons. These methods give the possibility not only of theoretical analysis and simulation of collisions between solitons but also of monitoring the stability and the general dynamics of non-integrable and non-linear models alike.

There is a number of studies in which the use of solitonic optical pulses for the purposes of carrying out computations has been investigated (Jakubowski et al. 1996, 2001; Bakaoukas and Edwards 2009a; Blair 1998). In this present paper only temporal solitons (involving a balance between Kerr type non-linearities and dispersive effects in glass fibres) are concerned. At this early point the fact that the interactions between solitons of this type can be a relatively long-range phenomenon need to be emphasised, because the Kerr non-linearity is a relatively weak effect.

In what follows in this introduction section, the discussion is focusing on the 3NLS equation domain. For a more extensive and thorough discussion the reader is referred to Bakaoukas and Edwards (2009b, 2013), Bakaoukas (2013, 2014, 2015) where the application of first order and second order solitons, following the Toffoli gates prototype as well as others, has been presented and verified regarding their computational abilities in terms of logic gates formations.

A positive value for “dispersion” parameter describes the formation of bright optical solitons whilst a negative value leads to the formation of dark solitons. The 3NLS equation in general, describes a modulated wave packet propagating through a non-linear dispersive medium with a constant velocity. For certain initial pulse shapes (the “reflectionless potentials”), the 3NLS equation is completely integrable and the evolution of a soliton can be found in closed form by means of the “Inverse Scattering Transform” (IST) (Ablowitz and Segur 1981). Solitons arising out of a balance between dispersive and Kerr non-linearity effects possess dominant characteristic features one of which is the elastic collisions between them. Solutions described by non-integrable non-linear wave equations on the other hand are usually referred to as “solitary waves” and collisions between solitary waves are inelastic and more complex in character. A solution of the integrable 3NLS equation applicable to pulse propagation in optical fibres is the hyperbolic secant where an arbitrary positive number representing the soliton order, the distance along the fibre, and time, all in normalised dimensionless units, are the main parameters forming the initial soliton propagation envelope. By coupling pulses in and out of a fibre at appropriate points (of distance and time), useful computation could be possible based on collisions between solitons within the fibre.

The material presented in Bakaoukas and Edwards (2009b), in particular, shows that in situations where optical solitons are formed within optical fibres (simulations have been carried out using all the above mentioned numerical techniques), with appropriate practical arrangements, computationally universal systems based on collisions between first order solitons are possible using logical gates based on the “controlled” type of gates originally proposed by Toffoli (1980), Fredkin and Toffoli (1981). As an extension to what presented in the above mentioned papers, in this present paper, the numerical study of collisions between first order solitons is expanded leading towards an all-optical Fast Fourier Transform (FFT) calculation. The CN and CCN soliton gates continue to be the essential ingredient of the computational model.

## Soliton collisions and computational scheme in the 3NLS equation domain

To be able to present and analyse the properties and the basic features of a soliton computational system in a domain described by the 3NLS Eq. (1), we need to present first the basic requirements for computation, which include: cascadability, fanout, and Boolean completeness. In general terms, cascadability requires that the output of one device can serve as input to another; fanout refers to the ability of a logic gate to drive at least two similar gates; and Boolean completeness makes it possible to perform arbitrary computation. The 3NLS equation domain system can be characterised as an “oblivious” system (one that is governed by totally elastic collisions). As we are about to see in what follows, “oblivious” soliton systems under certain conditions, can perform useful computations by a direct simulation of Toffoli logic gates.

1 $$\begin{aligned} \frac{\partial u}{\partial z} = -\frac{j}{2} { sgn}({\beta }_{2}) \frac{{\partial }^{2}u}{\partial {T}^{2}}+{B}\frac{{\partial }^{3}u}{\partial {T}^{3}}+{j}{\gamma }{|{u}^{2}|}{u}-{\Gamma }{u} \end{aligned}$$

Mathematically, a couple of solitonic pulses, which commences propagating down an optical fibre and possesses all the properties required to end up in a collision, can be described by the following equation:

2 $$\begin{aligned} u(0,\tau )\,=\, & {} r\ { sech}(r(\tau - {q}_{0})){e}^{j\theta }{e}^{jv\tau }\nonumber \\&+\, r\ { sech}(r(\tau + {q}_{0})){e}^{j\theta }{e}^{jv\tau } \end{aligned}$$

where, *r* represents the amplitude of the solitons, $\theta$ is the relative phase value, and ${q}_{0}$ is the initial displacement between the two solitonic pulses.

**Fig. 1:**
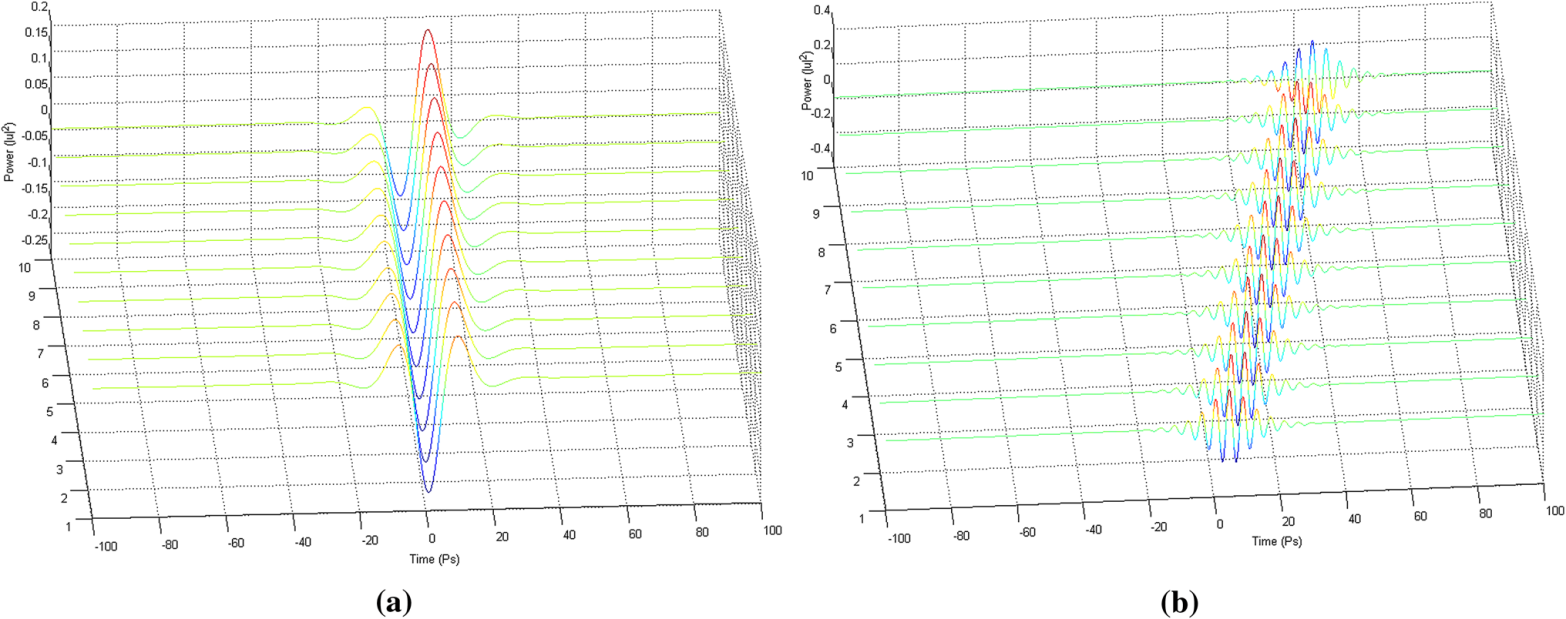
**a** Solitonic pulse carrier (the soliton velocity is equal to 0.3) and, **b** solitonic pulse carrier (the soliton velocity is equal to 1.5)

By solving the basic soliton equation using the IST we come to know that the velocity of a solitonic pulse depends on the frequency of the modulated carrier. So if we vary the frequency of the carrier we simultaneously alter the velocity of the solitonic pulse itself. The second exponential term in (2) does just that; alters the frequency of the carrier by a rate corresponding to the value given to its variable, and simultaneously alters that way the velocity of the pulse itself by the desired amount (Fig. 1).

**Fig. 2:**
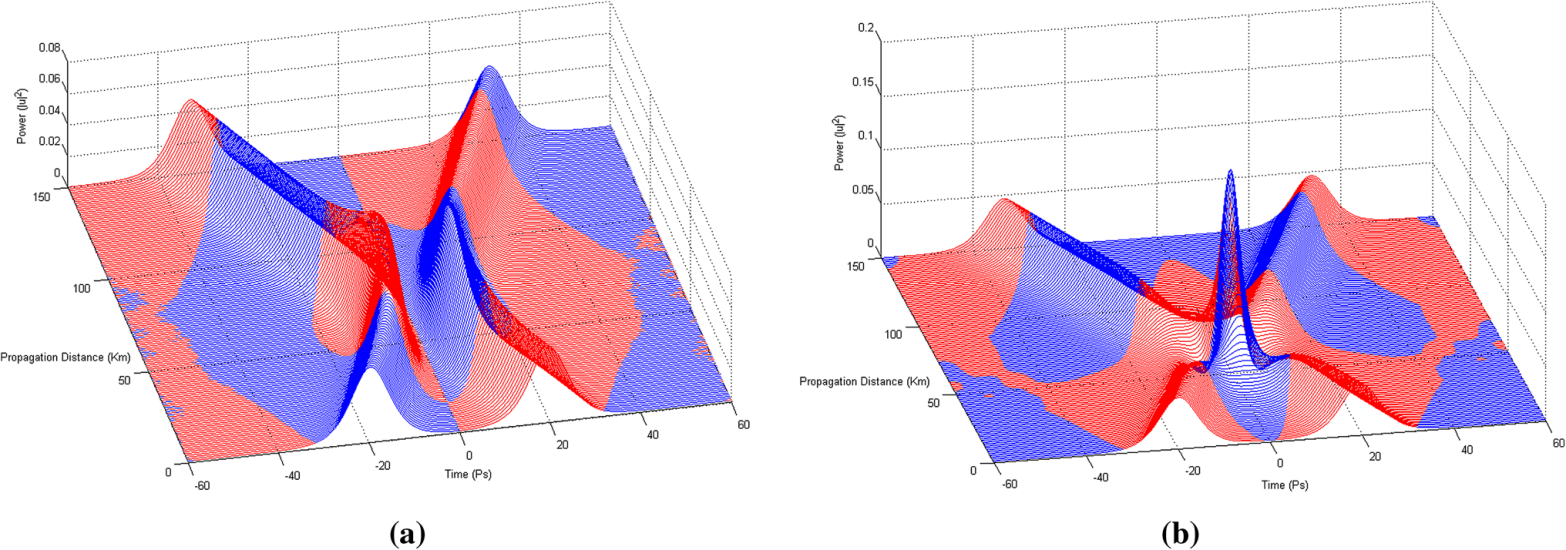
Collision between two first order solitons: **a** the solitons are out of phase and the repulsive force causes them to change their propagation direction (the soliton speeds are $+\,0.3$ and $-\,0.3$) and, **b** the solitons are in phase and the attractive force causes them to pass through each other maintaining their propagation directions (the soliton speeds are again $+\,0.3$ and $-\,0.3$). Maximum magnitude for the soliton envelope exactly at the point of collision is typical for this type of collision

**Fig. 3:**
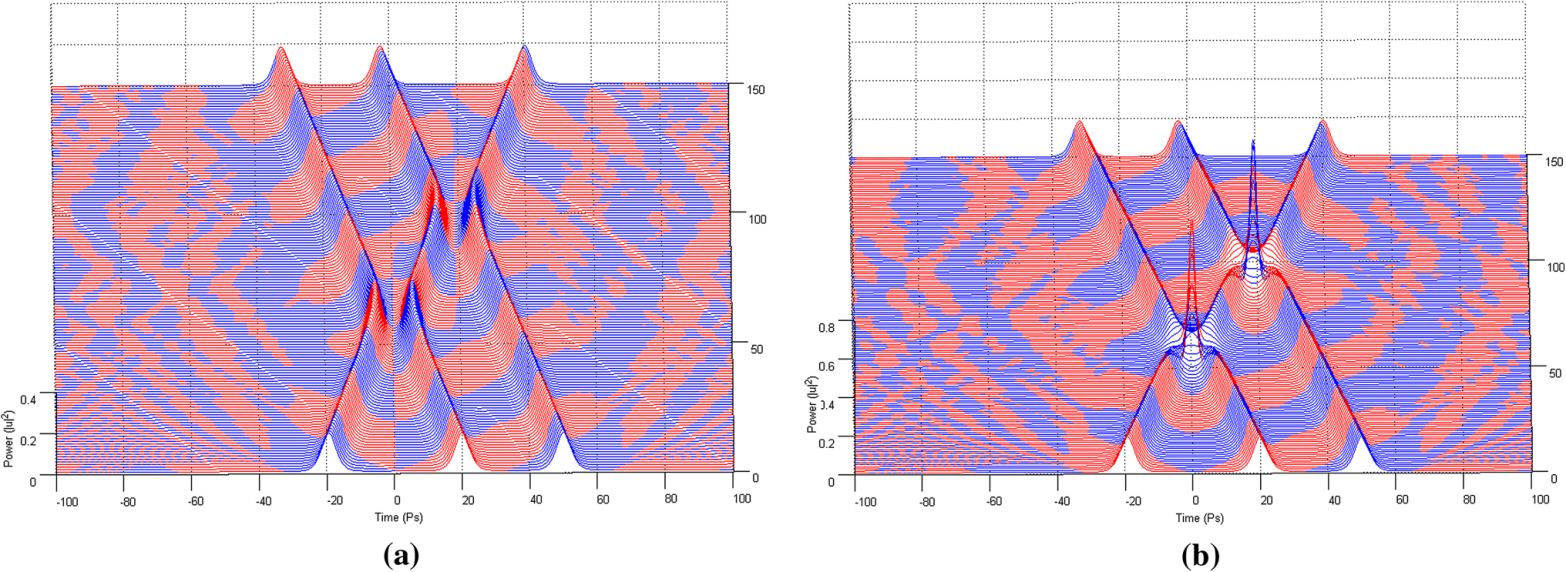
Collision between three first order solitons: **a** the first and the third soliton possess the same phase value, while the second soliton has a phase difference of $\pi$ in relation to the other two and, **b** all three solitons possess the same phase value

For the purposes of this paper only collision situations between two and three solitons (Figs. 2, 3) will be discussed and presented in details, since these are the fundamental building blocks out of which the 3NLS equation domain computational system is consisting of (interactions between solitons have been proved unable to offer computationally useful properties and soliton arrangements Bakaoukas 2014). In this scheme, two situations can be distinguished: (a) the solitons collide and are in phase, or (b) the solitons collide and are out of phase. In physical terms this can be translated as between the two solitons emerging an attractive or a repulsive force, respectively (Fig. 2). The presence and the strength of the repulsive or attractive force depends on the relative phase values of the two pulses.

As a first step into starting describing the fundamentals of the computational system itself now, the encoding rules for representing bits into the computation system will be set by initially admitting the existence of only two solitons in it. One soliton with a phase value of $\pi$ and one soliton with a phase value of 0. This way there can only strictly exist two types of collisions between solitons in the computational system: (a) two solitons collide and are in phase or, (b) two solitons collide and are out of phase (Fig. 2). Now the solitons can be directly used as input values to a solitons defined type of logic gate. The most important fact of all is that these two types of collisions inherently possess the property of sequencing, so they can be cascaded.

**Fig. 4:**
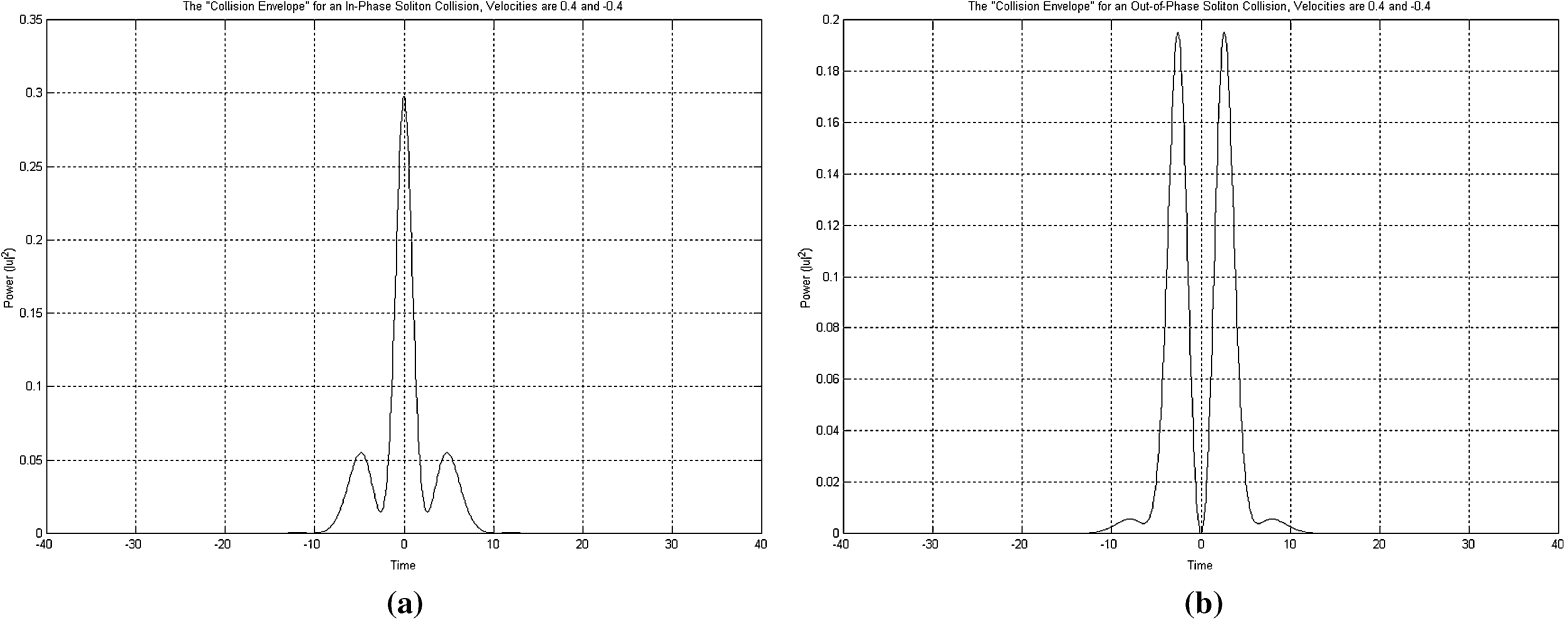
The collision envelope for two solitons in and out of phase. **a** The collision envelope for two solitons in phase. **b** The collision envelope for two solitons out of phase

**Fig. 5:**
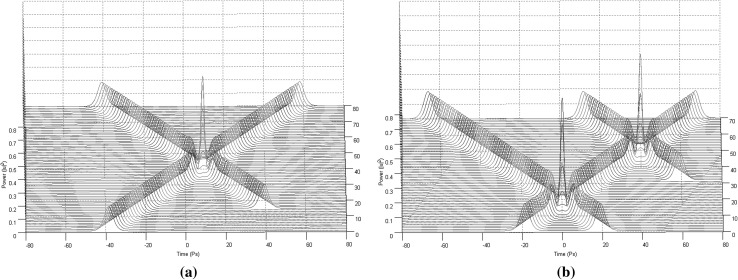
**a** A collision between two solitons. The second soliton is a “time-gated” input soliton and, **b** collision between three solitons. The third soliton taking part in the collision is a “time-gated” soliton in phase with the initial two

By using these rules things can be stretched a bit further and into safely considering collisions between solitons as the inner process of a solitons defined logic gate, with the initial solitons formulating the input to the logic gate values and the two solitons recovered with their original state intact after the collision, as formulating the output values of the logic gate. So, basically, we split the whole process of a collision into two important parts: (a) the logic gate length, bounded between the point at which solitons begin to propagate through the medium and that at which the two solitons emerge intact from the collision with time positions in reverse order, and (b) the point at which the two solitons collide, creating a characteristic for their phase values “collision envelope” (Figs. 3, 4). Introducing a third soliton into the arrangement, to which we refer to as “time-gated” soliton (Fig. 5) and is a generated by the system soliton with a phase value also determined accordingly by it. Effectively what is achieved here is within a system using three instead of two solitons per collision (logic gate), and by using appropriate combinations of phase values, the input solitons to be converted to control specific solitons and data specific solitons, achieving performing that way useful calculations.

Let us now see how we can use this three solitons arrangement and its properties as a full computational element (logic gate). In order to achieve this we assign to each soliton participating in the arrangement either the binary value 0 or the binary value 1, the way previously explained. The next step is to assume that the third soliton is a “time-gated” soliton with a phase value determined accordingly by the system as the process progresses. This practically means that the “time-gated” soliton does not take part in the calculation process from the start but is created and begins to propagate down the fibre at another specific point in time as a result of a decision (command) initiated by the system. The right point in time for this “time-gated” soliton to start propagating is defined as the one immediately following the collision between the two initial to the logic gate arrangement solitons. This forces the third (“time-gated”) soliton to play the role of the “controlled” by the system soliton. Within the boundaries of the computational system presented here, the phase value of the created (“time-gated”) soliton and its propagation timing are decided dynamically by the system after certain conditions have been taken into consideration, which in our case are the phase values of the two initial solitons. Now the arrangement can successfully simulate the CN and CCN Toffoli logic gates.

In setting the rules under which the system will be able to distinguish between conditions calling for the generation of a “time-gated” soliton with a phase value of 0 and those calling for the generation of a “time-gated” soliton with a phase value of *pi*, we assign a Boolean value to each one of the two envelope types resulting after a collision has taken place, and located at exactly the midpoint of the whole collision length (Fig. 4). The collision envelope occurring when two solitons collide and are in phase, is assigned the binary value 0, while the collision envelope occurring when two solitons collide and are out of phase is assigned the binary value 1. Thus, a system able to “read” the collision envelope within the mathematically determined collision point of the two initial solitons (input solitons to the logic gate arrangement), is also capable of recognising the nature of the collision, i.e. to recognise immediately whether the two solitons participating in the collision were in phase or out of phase. Finally, using these assumptions, in conjunction with the “time-gated” solitons concept, we can successfully simulate the operation of a CN Toffoli logic gate in the 3NLS equation domain and build a truth table for this type of gates (Bakaoukas and Edwards 2009a).

## Numerical methods for soliton propagation simulation

In this section, the numerical techniques used for obtaining the simulation results are briefly described and example outputs are presented of using them. The recognition that the mathematical complexity of soliton solutions arises only because of the dependency of the refractive index *n* on spatially varying intensity could eradicate the complexity if, instead, *n* could be defined as depended not on a spatially varying intensity but on the total beam power. This can be achieved in a heuristic model by assuming that the medium has a non-local response with a correlation length much larger than the beam diameter. The non-linear wave equation then becomes linear and readily solvable, but the solution for solitons still contains the essential characteristic features. Physically, the model transforms the problem into a simple case of linear propagation of thin beams in a waveguide. With the assumption that the beams always stay close to the axis, the refractive index makes the wave equation identical to the “Time-Dependent Schrödinger Equation” (TDSE) for a linear harmonic oscillator, the solution of which is well known to all physicists. The physics of solitons can then be readily appreciated.

### Finite difference Runge–Kutta technique (FDRKT)

Despite the complicated mathematical analysis and the advanced mathematical techniques (such as IST) someone can employ numerical techniques as well in order to analytically obtain solutions to the 3NLS equation. The equation itself is but a partial differential equation, which, by applying appropriate initial and boundary conditions can be solved by one of the available numerical techniques. The most popular, mainly because of the high accuracy of the Runge–Kutta technique involved, is the “Finite Difference Runge–Kutta Technique”. To apply the finite difference Runge–Kutta technique we need first to express the derivatives as a set of values representative of the continuous function:

3 $$\begin{aligned}&\left\{ \begin{array}{c} f(x + {\Delta }x) = f(x)+\frac{{\Delta }x}{1!}\frac{\partial {f(x)}}{\partial {x}}+\frac{{\Delta {x}}^{2}}{2!}\frac{{\partial }^{2}f(x)}{\partial {{x}^{}2}}\\ f(x - {\Delta }x) = f(x)-\frac{{\Delta }x}{1!}\frac{\partial {f(x)}}{\partial {x}}+\frac{{\Delta {x}}^{2}}{2!}\frac{{\partial }^{2}f(x)}{\partial {{x}^{}2}} \end{array}\right\} \nonumber \\&\Rightarrow \frac{{\partial }^{2}f(x)}{\partial {{x}^{2}}} \,=\, \frac{f(x + \Delta {x}) - 2f(x) + f(x - \Delta {x})}{\Delta {{x}^{2}}} \end{aligned}$$

**Fig. 6:**
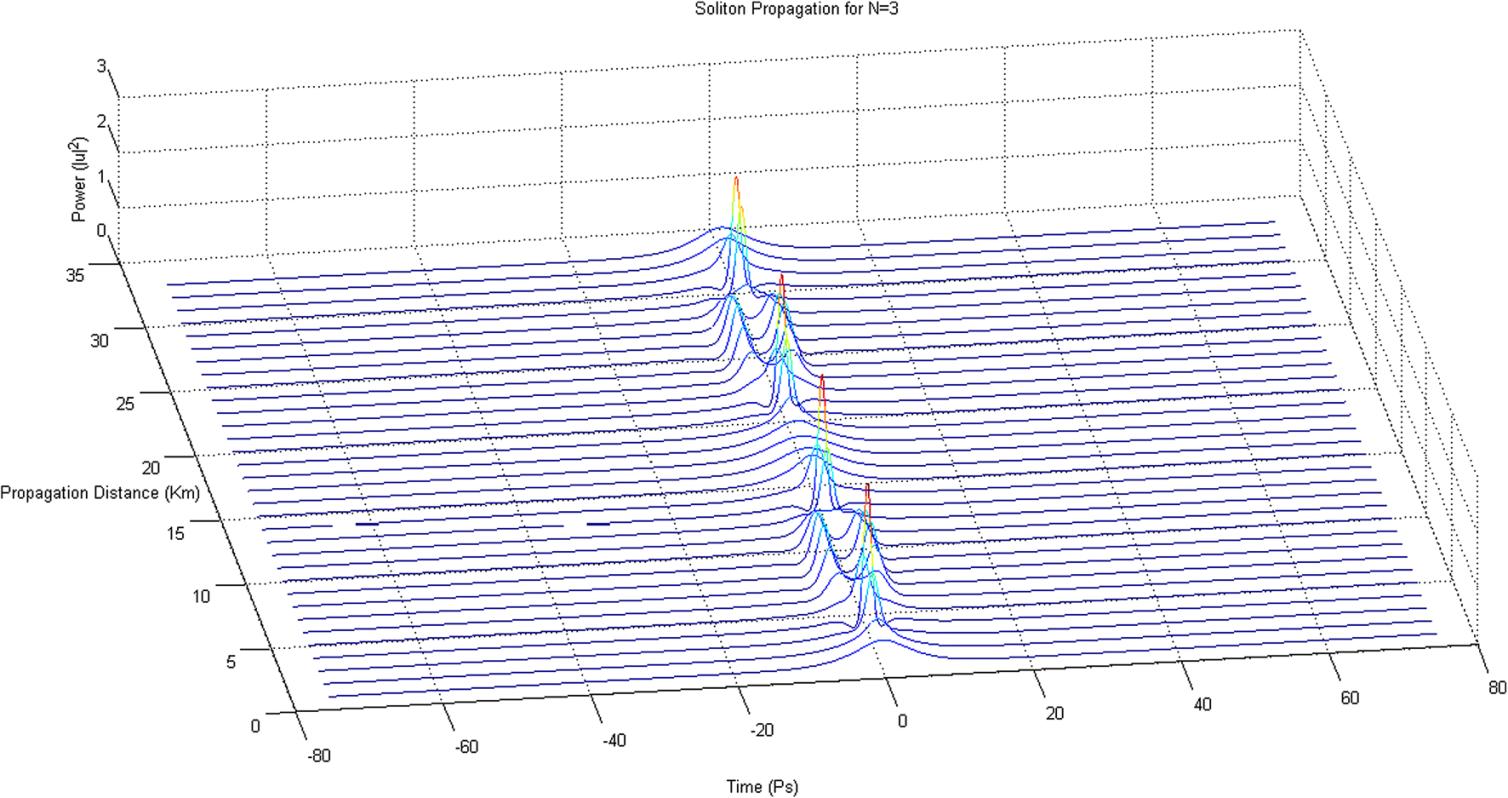
Third-order soliton propagating down a length of fibre (“Finite Difference Runge–Kutta Method”)

In the 3NLSE we substitute the second-order dispersion driven second derivative (and/or the third-order dispersion driven third derivative) with its finite difference equivalent and we obtain a form of the equation directly solvable by means of the Runge–Kutta technique (Fig. 6).

### Split-step Fourier transform (SSFT)

The “Split-Step Fourier Transform” (SSFT) is considered, well up to these days, the technique of choice for solving the 3NLSE and simulating the propagation of optical solutions for a variety of physical parameters and conditions. However, needs to be emphasised that the SSFT method will not work well for simulating situations where there exists a forward and backward propagating wave.

A closer look in (1) reveals that the dispersion and the non-linearity are decomposed. Taking advantage of this fact the 3NLS equation can be solved relatively easily through the SSFT method. In fact, the technique has taken its name exactly because of this separation between dispersion and non-linearity. The first step is to make use of the operators $\hat{D}$ and $\hat{N}$ to correspond to the dispersive and non-linear terms respectively, so (1) can take the form:

4 $$\begin{aligned} \frac{\partial {U}}{\partial {z}}\,=\,{(\hat{D}+{\hat{N}})}{U} \end{aligned}$$

Assuming now that only each one of them operating we can obtain:

5 $$\begin{aligned} {\hat{D}}& = {{U}_{1}}(z,t) \\&= { IFFT}\left({exp}\left( \left( {\frac{j}{2}{\beta }_{2}{\omega }^{2}-\frac{j}{6}{\beta }_{3}{\omega }^{3}-\frac{a}{2}}\right) z\right) { FFT}({U}(0,t))\right) \end{aligned}$$

6 $$\begin{aligned} {\hat{N}}&={U}(z,t)={U}_{1}(z,t){ exp}\left( {j\gamma {P}_{0}{|{U}_{1}(z,t)|}^{2}z}\right) \end{aligned}$$

At this point we need to note that the $\hat{N}$ operator multiplies the field solution and is a function of the solution *U*(*z*, *t*), while the operator $\hat{D}$ is a differential operator expressed in terms of time derivatives that operate on *U*(*z*, *t*). In order to make the computation more efficient the calculation of $\hat{D}$ is performed in the frequency domain with the result of transforming the derivatives in the time domain to a simple multiplication in the frequency domain.

**Fig. 7:**
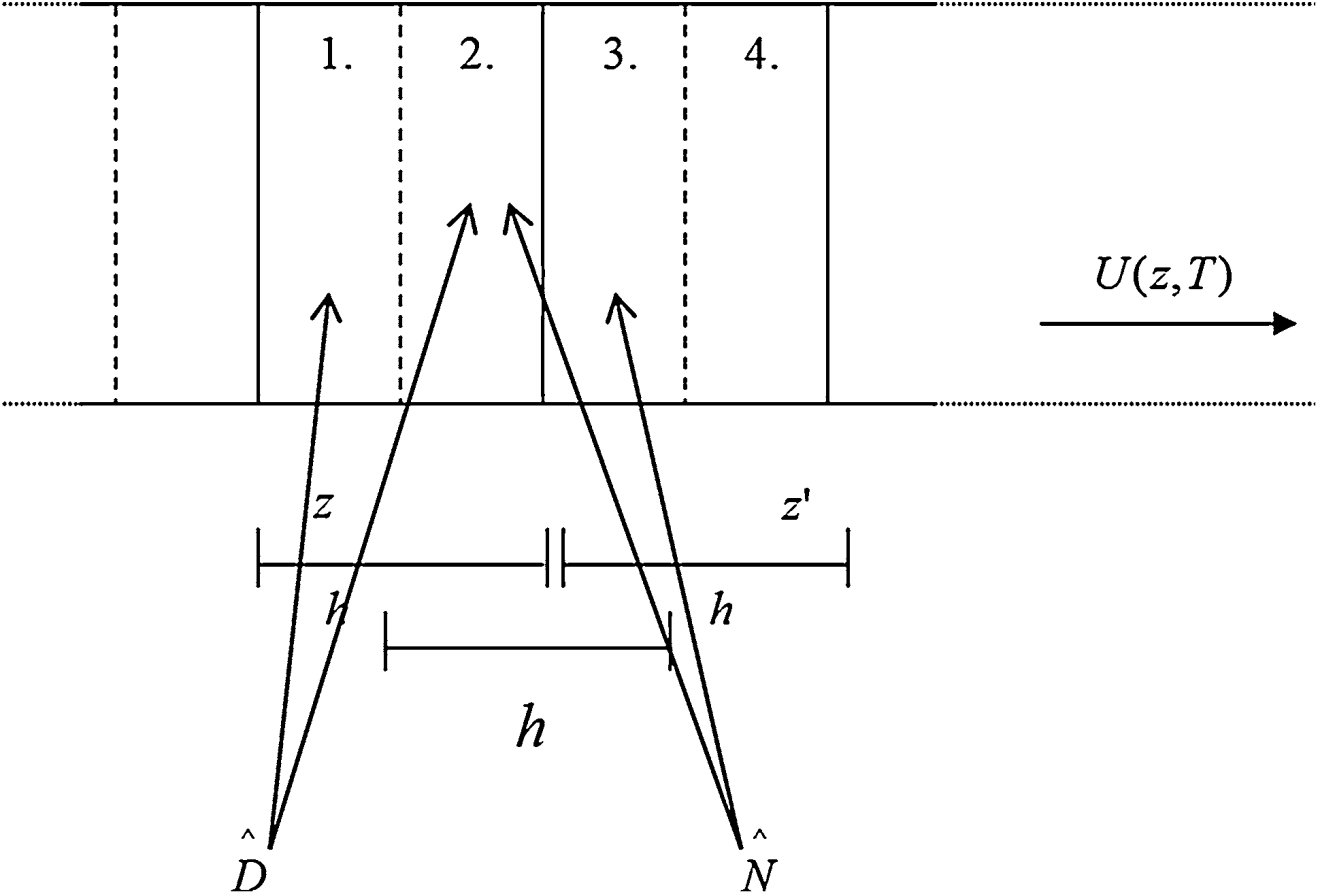
The SSFT method for one iteration (the length of every step is *h*)

The SSFT method is an iterative method that determines the field solution for special steps of *h*. This is performed in a step-by-step approach and lasts for the entire length of the fibre. A length of optical fibre *L* is broken down into ${S}_{L}=\frac{L}{h}$ steps of length *h* (Fig. 7). So, applying the SSFT method requires the following iterative procedure:

1. The total fibre length is divided into a number of segments of length *h*.
2. At the beginning of each of them we compute the *FFT* of the every time initial condition *U*(0, *t*).
3. The pulse is propagated in the frequency domain for a distance $\frac{h}{2}$ under the effect of dispersion only.
4. At the middle of the segment, the *IFFT* is applied in order to come back to the time domain and calculate the contribution of non-Linearity (and if desired fibre loss) in the whole segment.
5. In the last step another *FFT* is evaluated to return to the frequency domain and propagate the field through the remaining distance $\frac{h}{2}$, with dispersion only.
6. The results obtained are used as the initial condition for the following segment and the process is repeated until the total fibre distance is achieved.

The initial condition or pulse shape is necessary to start the calculation. After propagation over a distance of *h* in both the linear and non-linear parts, the results can be used as the initial condition for a further propagation distance of *h*. This process is repeated until the required overall propagation distance is achieved.

**Fig. 8:**
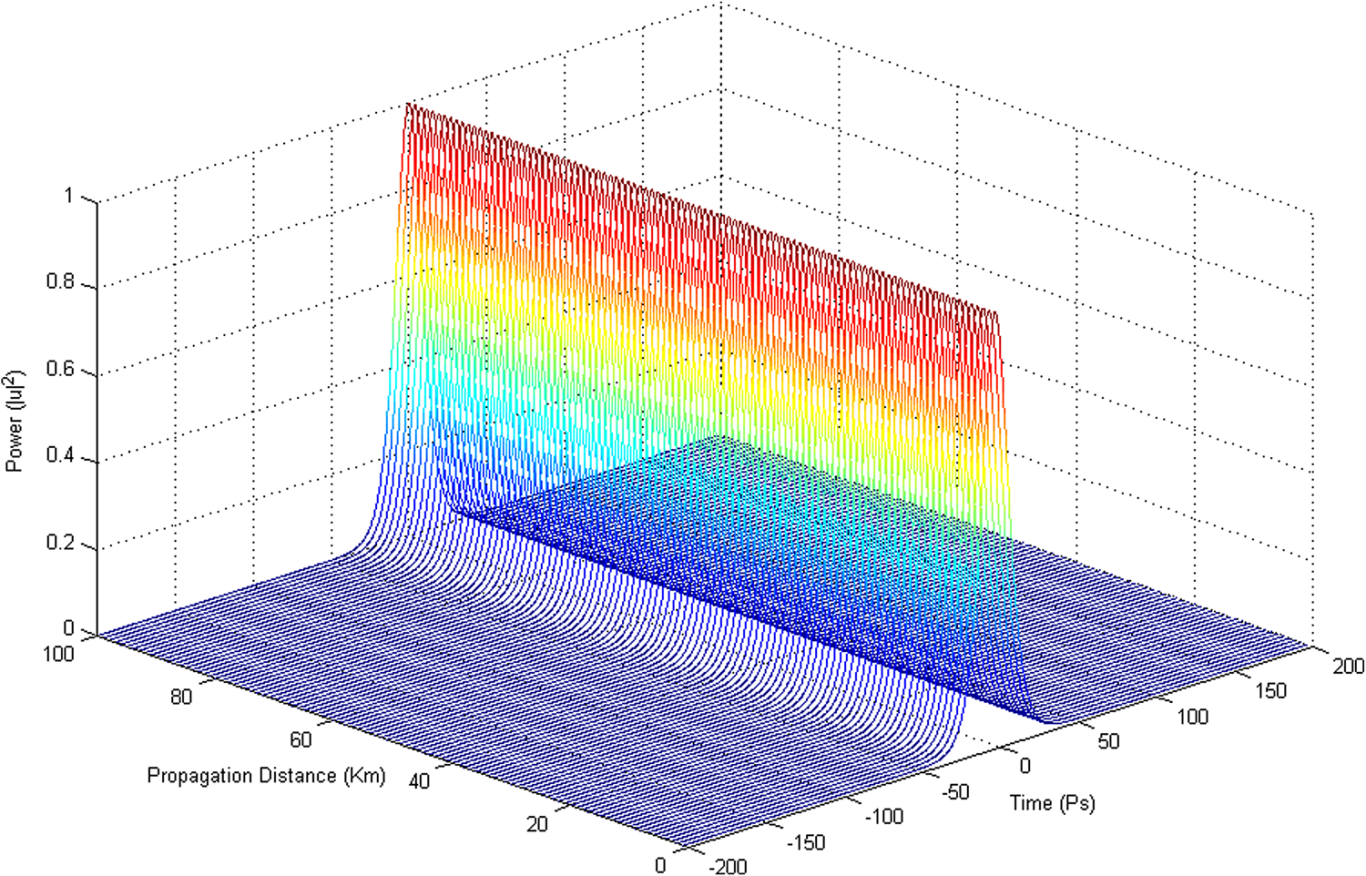
A first-order soliton pulse in the 3NLS equation domain. The chromatic dispersion effect blends perfectly with the Kerr non-linearity effect and the result is the creation and propagation of a solitonic pulse. For simulating the soliton propagation the SSFT method was used

Considering the initial fundamental optical fibre parameters: $h = 0.01$ Km, ${\beta }_{2} = -3\,\,{\frac{{\mathrm{ps}}^{2}}{\mathrm{Km}}}$, $\gamma = 2{\,\,\frac{1}{\frac{\mathrm{W}}{\mathrm{Km}}}}$, $Full\ Soliton\ Width\ (FSW) = 10$ ps, $Soliton\ order = 1$, soliton propagation down the fibre using the SSFT method is achieved (Fig. 8).

As a final remark the *FFT* algorithm imposes restrictions on the sample array format of a nature that cannot be ignored. The sample array of *U*(*z*, *t*) for each value of *z* must have $N={2}^{n}$ points as required by the *FFT* algorithm. The initial array *U*(0, *t*) must sample the initial pulse $\Delta {t}$ with adequate temporal resolution and be temporally wide enough to prevent aliasing and wrapping errors. Of course, as is the case for all similar situations the sampling rate is to be given by the Nyquist theorem.

In contrast to what said above in regards to $\Delta {t}$, there is no strict mathematical restriction on the step-size *h* for the *SSFT* method other than choosing a very small *h* will result in a very accurate but very computationally time demanding simulation. Also, we need to have in mind that choosing *h* smaller than the carrier wavelength ${\lambda }_{0}$ is physically meaningless. On the other hand choosing *h* to be too large does not conserve spectral energy. The maximum choice of the step-size depends on the specific dispersive and non-linear properties.

### Fourier series analysis technique (FSAT)

**Fig. 9:**
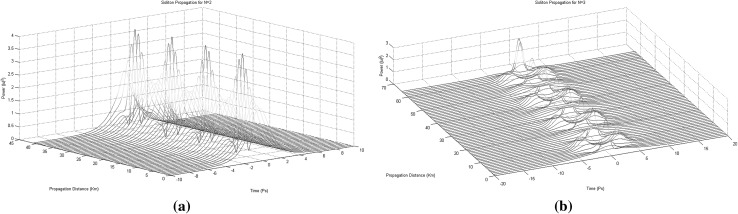
Second-order and third-order solitons as simulated using the FSAT numerical method. **a** A second-order soliton propagating down a length of fibre (FSAT using the MATLAB™ODE45() function). **b** Third-order soliton propagating down a length of fibre (FSAT using the Runge–Kutta method)

One of the commonly used set of techniques for solving “Partial Differential Equation” (PDE) systems is the “separation of variables” set, of which the “eigenfunction expansion” technique is the most representative example, in which a solution of the following form is assumed:

7 $$\begin{aligned} u(x,t)={\sum \limits ^{\infty }_{n=1}}{{a}_{n}(t)}{{\phi }_{n}(x)} \end{aligned}$$

where, ${\phi }_{n}(x)$ are an orthogonal set of eigenfunctions and we have assumed that the PDE is now described by a scalar quantity *u*(*x*, *t*). The ${\phi }_{n}(x)$ can be any orthogonal set of functions in which ${\delta }_{jk}$ is the Dirac function and $({\phi }_{j},{\phi }_{k})={\int }{\phi }_{j}{\phi }^{*}_{k}{\partial {x}}$ gives the inner product (Fig. 9).

Depending on the physical problem at hand, the two most common eigenfunctions used are the Fourier series and the Chebyshev polynomials, mainly because of their good correlation with the majority of physical phenomena, their spectral accuracy properties, and computational speed.

In order to solve the 3NLS equation computationally, a Fourier series expansion is used, thus, use can be made of the standard *FFT* technique. The “Fourier Series Analysis Technique” initially expresses the pulse envelope function in terms of a Fourier series in which $\hat{u}_{n}(x)$ is the Fourier amplitude coefficient, and $\epsilon$ the fundamental frequency. This procedure yields:

8 $$\begin{aligned} {\frac{\partial {{\hat{u}}_{n}(x)}}{\partial {x}}}=[-j{\sigma }(n)-{\Gamma }]{{\hat{u}}_{n}}(x)+{j{N}^{2}}{{\sum \limits _{\forall {\mu -\nu +\lambda =n}}}}{{\hat{u}}_{\mu }(x)}{{\hat{u}}^{*}_{\nu }(x)}{{\hat{u}}_{\lambda }(x)} \end{aligned}$$

where *n* is an integer ${-M}\le {n}\le {M}$ and ${\sigma }(n)={{n}^{2}\frac{{\epsilon }^{2}}{2}+B{n}^{3}{\epsilon }^{3}}$. The equation represents a set of $2M+1$ “First-order Partial Differential Equations” (FPDE) of complex variable, which can be separated into its real and imaginary parts, and solved by a fourth-order Runge–Kutta method. The $2M+1$ initial conditions can be obtained for $x=0$. The final solution is in the time domain. Significant parameters of this method include the time window $\Delta {T}$, within which the signal is sampled for the Fourier series representation, the step length for integration $\Delta {x}$, and the integer *M*, related to the total number of samples $(2M+1)$.

### Fuzzy mesh analysis technique (FMAT)

The main advantage of this technique is its ability to allow for variation of the mesh size with the shape of the soliton pulse along the propagation distance, such that: (a) the calculation efficiency can be enhanced, and (b) the number of sampling points required can be greatly reduced. This technique requires for the soliton equation to be solved by first splitting it into two simpler parts which can be calculated easily, either analytically or numerically, in the time domain. In addition to that, the mesh size is controlled by the shape of the soliton pulse such that the number of sampling points used is in every propagation step minimised.

**Fig. 10:**
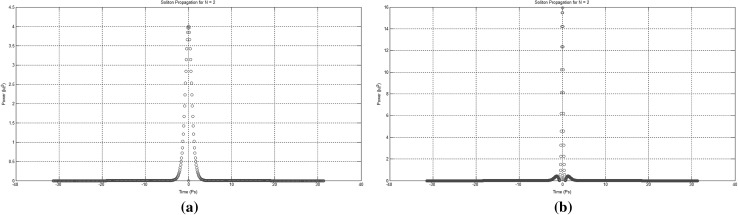
The two initial stages of the soliton envelope as simulated using the FMAT method. **a** Second-order soliton intial envelope. **b** Second-order soliton envelope after propagating a quarter period down the length of fibre

In order to implement the “Fuzzy Mesh Analysis Technique”, Eq. () is split into two parts, the linear and non-linear parts. It is obvious that any error arising as a result of the splitting operator is proportional to the choice of $\Delta (x)$, the propagation step. The non-linear part can be solved analytically in the time domain, and the linear part can be calculated by means of the “Finite-Element Analysis Technique” (Shum and Yu 1998). Summarising, the technique in separate computational steps can be arranged as follows:

1. The initial input pulse shape is set for computation.
2. The non-linear part is solved analytically in the time domain for a propagation distance of $\frac{\Delta (x)}{2}$.
3. $u(x + \frac{\Delta (x)}{2}, T)$ as obtained from step (2) is used as the initial condition for solving the Linear part for another propagation distance of $\frac{\Delta (x)}{2}$.
4. Mesh control is adopted each time after the calculation of the linear part such that the sampling profile can be optimised for the next calculation.
5. $u(x + \Delta (x), T)$ as obtained from step (3) is used as the initial condition for solving the non-linear part of another propagation distance provided that the propagation distance is not reached.
6. Steps (2) to (5) are repeated until the required propagation distance is reached.

**Fig. 11:**
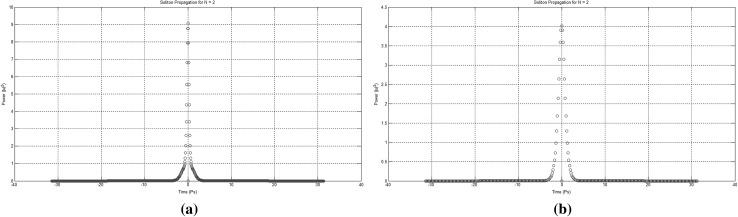
The two final stages of the soliton envelope as simulated using the FMAT method. **a** Second-order soliton envelope after propagating half a period down the length of fibre (FMAT method). **b** Second-order soliton envelope after propagating for a full period down the length of fibre (FMAT method)

The computational procedure of the algorithm for the mesh control, in individual steps is as follows:

1. Using cubic shape functions, the temporal soliton pulse shape at a particular propagation distance is deduced as a function of *T*.
2. Based on the calculated cubic shape functions, both the temporal pulse shape and slope of *u*(*x*, *T*) are obtained. Hence, the turning points ($\frac{\partial {u}(x, T)}{\partial {T}} = 0$) can be located by comparing the variation of the slope of *u*(*x*, *T*).
3. The distribution of sampling points is defined within a sampling window along the axis. The left and right boundaries of this sampling window are defined as the magnitude of *u*(*x*, *T*) just below ${10}^{-3}$. Based on the location of turning points, new values of mesh sizes as well as ${a}_{i}$ are assigned.
4. The procedures of assignment of mesh sizes can be described as follows: (a) The number of turning points ${N}_{tp}$ is counted within the sampling window, (b) The sampling window is subdivided into ${N}_{tp} + 1$ regions with the turning points as the boundaries of each subdivided region, (c) The sampling points within each subdivided region are equally spaced, but the number of sampling points ${N}_{j}$ can be different between subdivided regions, (d) If ${u}_{pj}$ and ${u}_{tj}$ are the boundaries of *u*(*x*, *T*) within a subdivided region, ${N}_{j}$ can be defined as ${N}_{j} = {R}_{j}({u}_{pj} {u}_{tj})$, where ${R}_{j}$ is a tuning factor. ${R}_{j}$ is adjusted such that the summation of sampling points within each subdivided region is equal to ${N}_{tp}$, and (e) If the total number of sampling points assigned to each subdivided region is more (or less) than the available sampling points of the sampling window, the above procedures are repeated for a different ${R}_{j}$ until the optimal mesh size is achieved.
5. Hence, new values of *u*(*x*, *T*) can be calculated using the optimised node distribution ${a}_{i}$.

Using this mesh control, more information of the soliton pulse can be obtained but without increasing the total number of sampling points (Figs. 10, 11).

## The half-adder processor scheme

The half-adder processor scheme, first introduced in Bakaoukas and Edwards (2009b), forms the essential central building block on which the overall FFT soliton computational scheme is wrapped around. The system reads the collision envelopes at distance and time specified points and uses this information to generate solitons with an appropriate phase value to represent the output of each “gate”. The phase values of two of the output solitons determine the “sum” and “carry” outputs at the end of the computation process whilst all other solitons are superfluous to this calculation. By definition the half-adder (the sum implementation) is given by:

9 $$\begin{aligned} \overline{\overline{\left( X \cdot \bar{Y}\right) } \cdot \overline{\left( \bar{X} \cdot Y\right) }} \end{aligned}$$

**Fig. 12:**
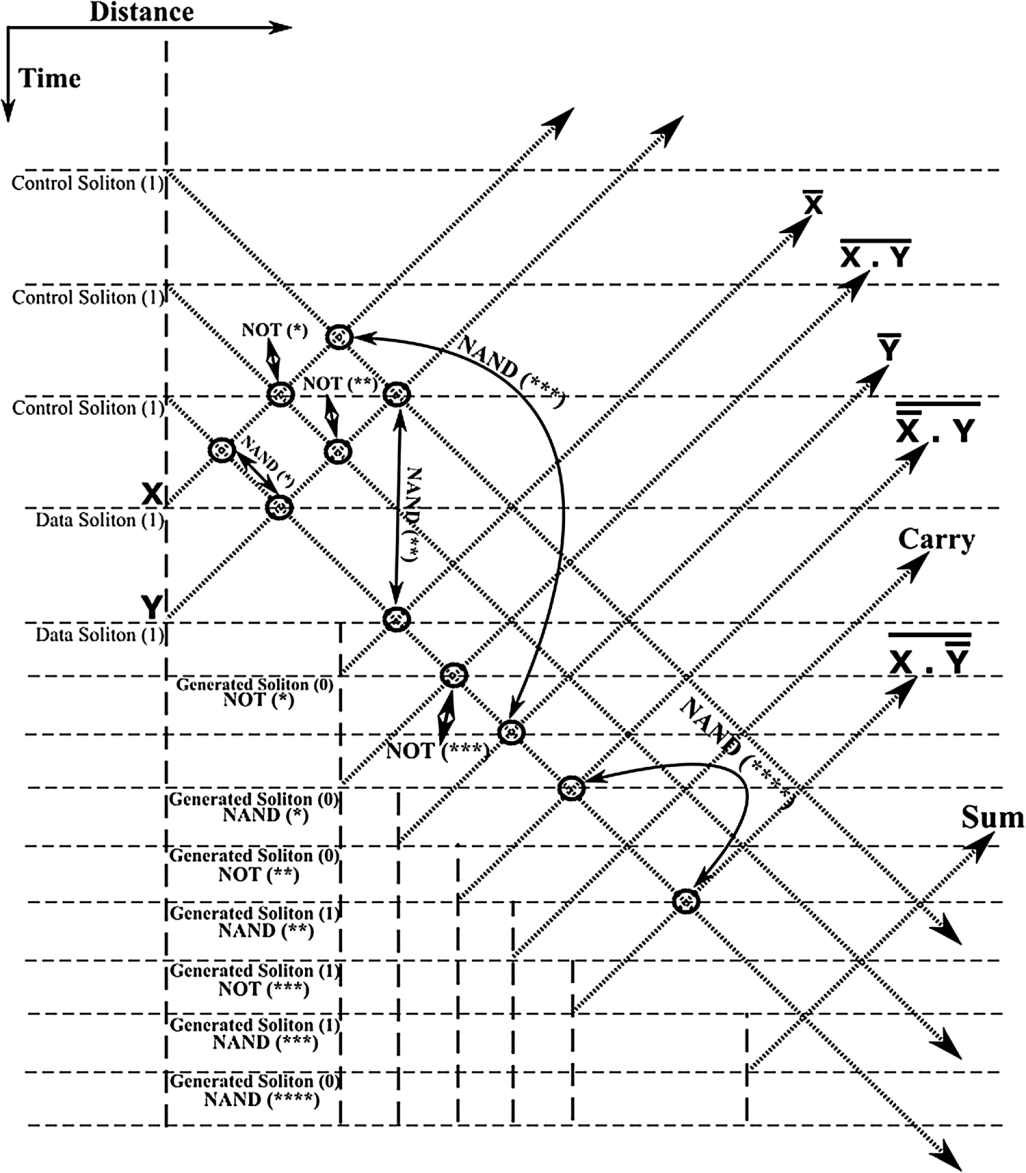
The half-adder processor

In Fig. 12 the equivalent soliton scheme, originally presented in Bakaoukas and Edwards (2009b), is reproduced here for convenience. The points highlighted in this schematic representation by means of a bold circle indicate functional points at which a soliton collision, part of a gate, takes place; while, X and Y denote the initial input data. Full “gate” arrangements have been named and numbered (e.g. NAND (*), indicates the first NAND in the computational arrangement, NAND (**) the second, etc.).

**Fig. 13:**
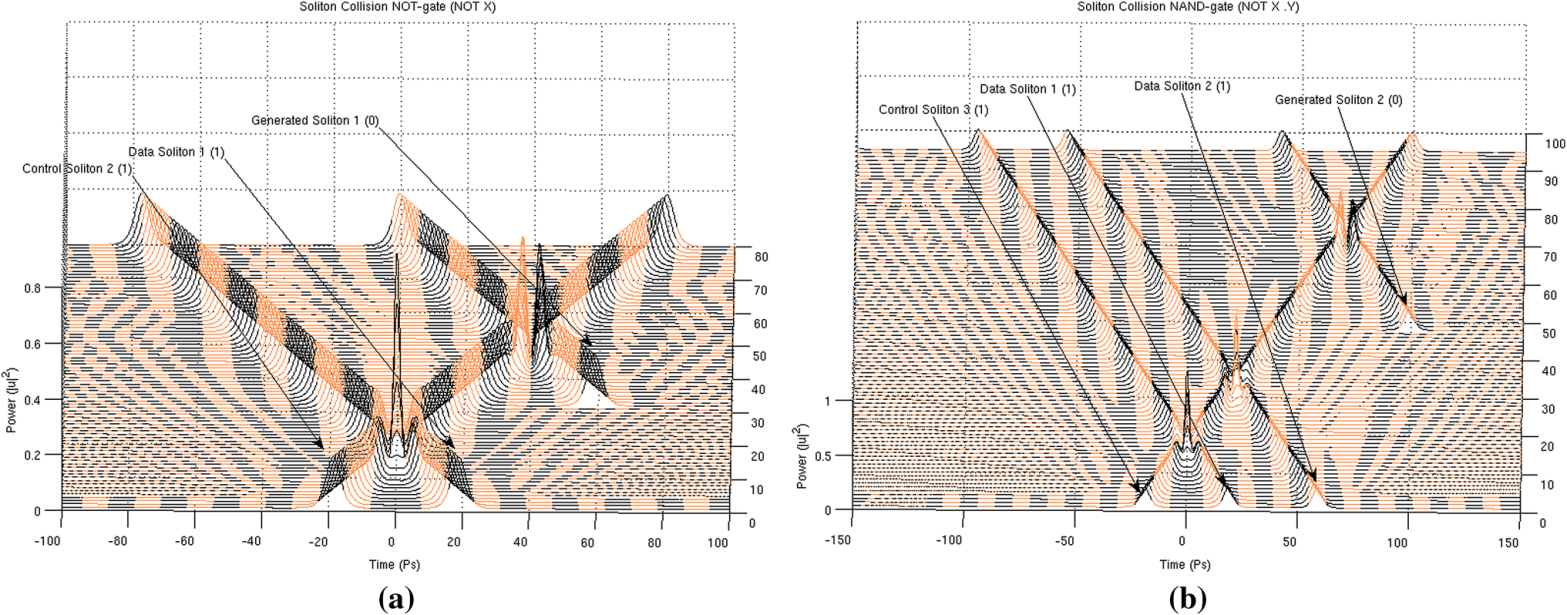
**a** The soliton “gate” NOT(*). The number in the brackets next to each soliton description is the bit value carried by the soliton and **b** the soliton “gate” NAND(*). The number in the brackets next to each soliton description is the bit value carried by the soliton

**Fig. 14:**
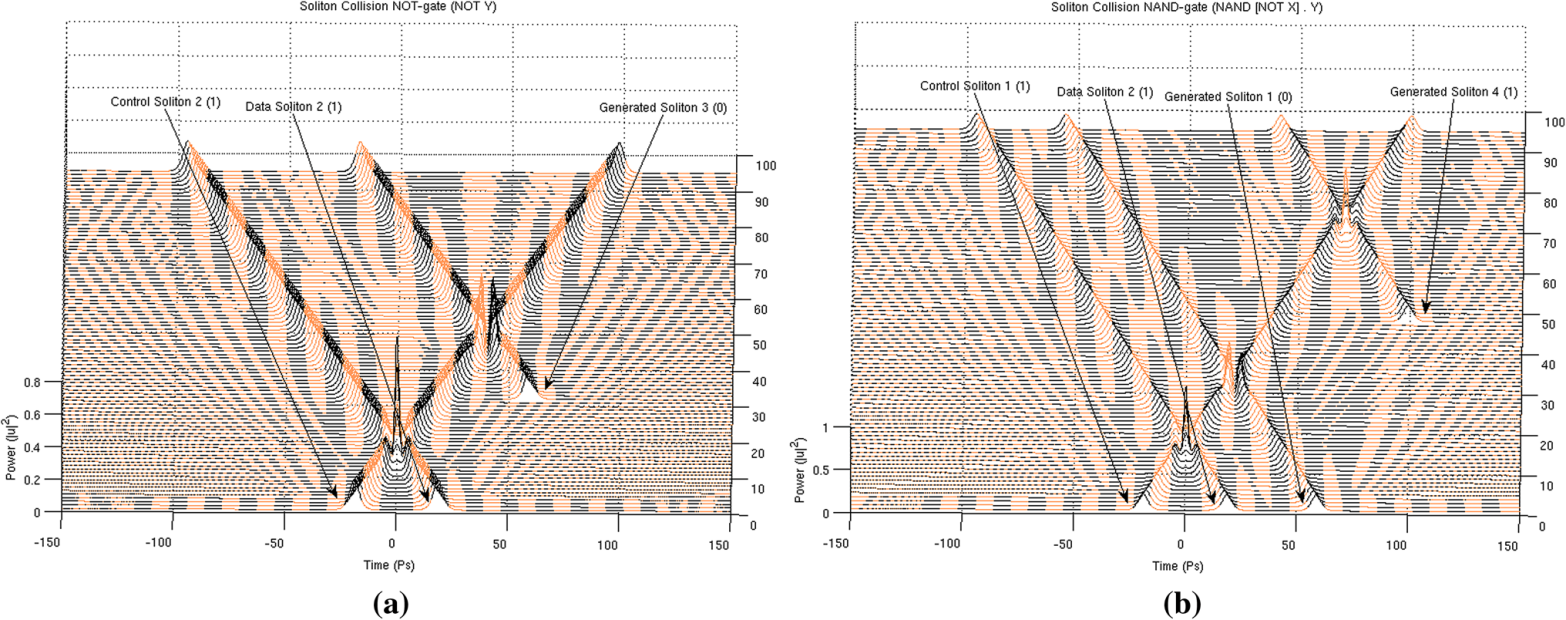
**a** The soliton “gate” NOT(**). The number in the brackets next to each soliton description is the bit value carried by the soliton and **b** the soliton “gate” NAND(**). The number in the brackets next to each soliton description is the bit value carried by the soliton

**Fig. 15:**
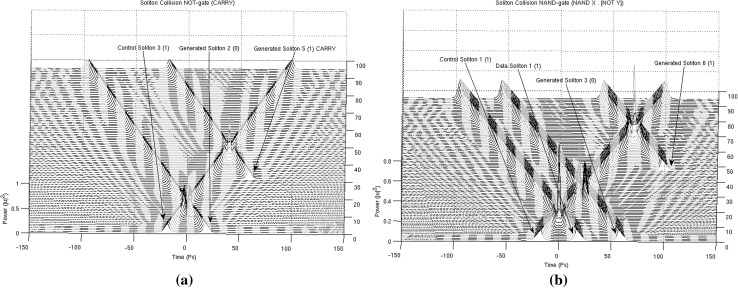
**a** The soliton “gate” NOT(***). The number in the brackets next to each soliton description is the bit value carried by the soliton and **b** the soliton “gate” NAND(***). The number in the brackets next to each soliton description is the bit value carried by the soliton

**Fig. 16:**
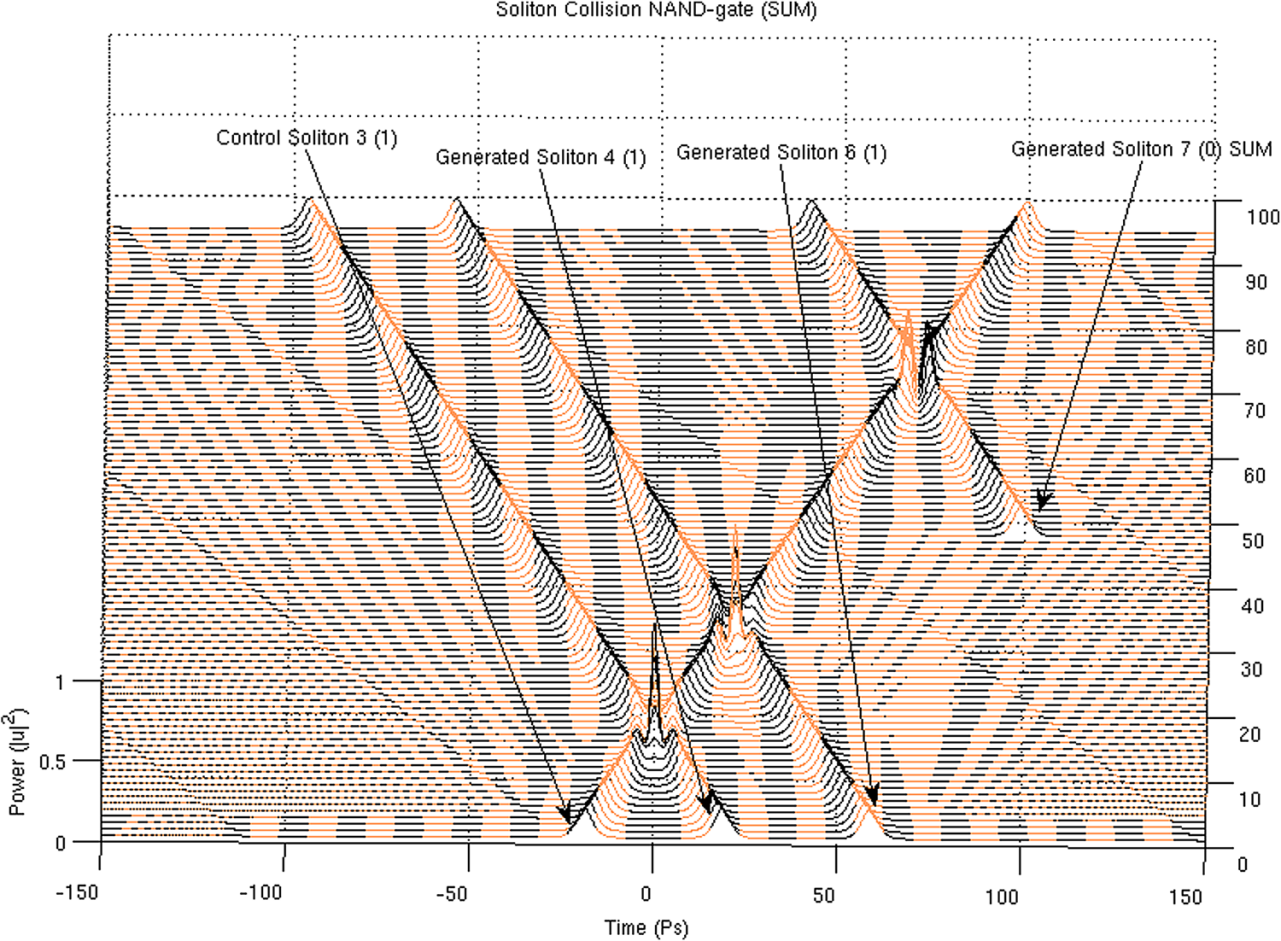
The soliton “gate” NAND(****). The number in the brackets next to each soliton description is the bit value carried by the soliton

In Figs. 13, 14, 15 and 16, the schematic representation of Fig. 12 is reflected on actual soliton collision simulations. Each individual gate-soliton collision is presented in a separate figure for clarity and comparison purposes. The simulation figures are to be followed in a top-to-bottom approach in the schematic representation of Fig. 12.

In all the figures the input–output “gate” sequence follows the soliton propagation direction. The point at which the soliton propagation begins (point 0 in the propagation scale across the depth of the figure) also reflects the input side of the “gate” and respectively, the point at which the soliton propagation ends (point 100 in the propagation scale across the depth of the figure) reflects the output side of the “gate”.

At this point and for the approach used for the presentation of the material to follow in this paper to become clear, we need to stretch-out the fact that the computational complexities involved are extensively simplified if can become apparent that the scheme is flexible enough to be gradually get “packed” in fixed-purpose calculation lengths. This approach doesn’t suppress the system from its generalisation properties, as the fixed reading points (as these have been identified and introduced in Bakaoukas and Edwards 2009a, b) still hold their properties and continue to provide the system with all the capabilities initially identified as inherently characteristic of the computational system at hand.

## The two 2-bit numbers multiplier

In this section we present the “Two 2-bit Numbers Multiplier”, which involves a half-adder as its lying-in-its-heart functional unit (“Three-bit Adder Arrangement”). The particular arrangement forms the compact small-scale equivalent of the “Two maximum-number-of-bits Numbers Multiplier”, which for general purpose calculations must involve full-adders as well as half-adders in its arrangement. The reason behind choosing the two 2-bit numbers multiplier is only the fact that the particular arrangement possesses all the functionalities and properties need to be demonstrated, while at the same time gives us the ability to keep the material presented at a minimum of extension and complexity in this paper.

**Fig. 17:**
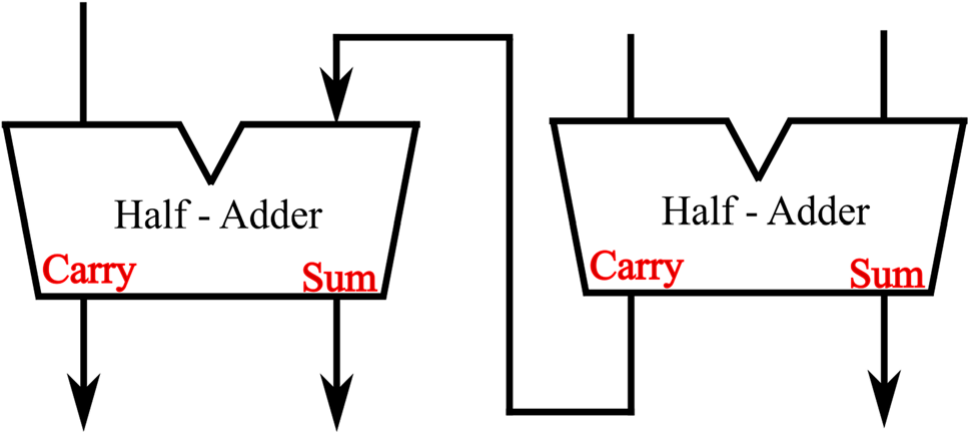
The three-bit adder

Starting from the half-adder arrangement, if we now take a closer look in Fig. 12 we will notice that all the output solitons need to be ignored after reading and only the output soliton representing the “carry” value is to be allowed to propagate further on and enter the cascading second half-adder arrangement. Is exactly this soliton-bit that is required for the arrangement to complete the three-bit adder arrangement output calculation as presented in a conventional block diagram in Fig. 17.

This “soliton suppression” requirement at the very end of a computational arrangement is not characteristic only of the computational scheme here presented but rather a common characteristic requirement in soliton computational arrangements as, for example, of the one introduced in Rand and Steiglitz (2007), where the additional property of not intersecting (solitons crossing paths but not colliding) is also a vital system characteristic requirement. The usual formal term coined for such kind of solitons is “Garbage Solitons” and is chosen to emphasise the fact that these solitons are to play no active role in the cascading calculations following the output of an arrangement. The way this soliton suppression can be physically achieved is, in general terms, a technicality, requiring some hands-on experimental work, in order for different methods and their corresponding effects on the overall computational arrangement to be properly studied. For these reasons we postpone, at this point, the explanation of how this soliton suppression can be accomplished.

**Fig. 18:**
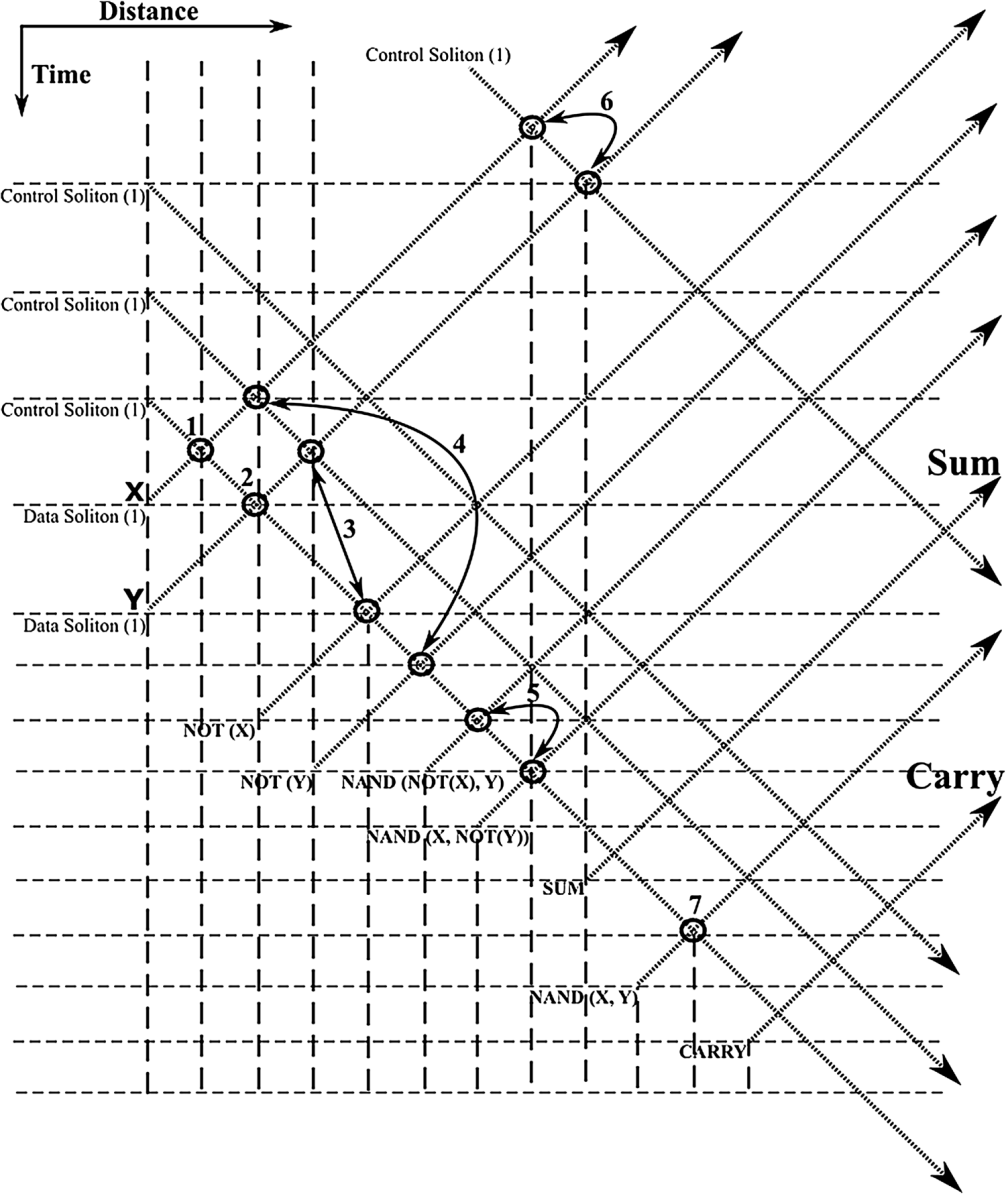
The alternative half-adder arrangement. [Logic gates: (1) NOT, (2) NOT, (3) NAND, (4) NAND, (5) NAND, (6) NAND, (7) NOT]

**Fig. 19:**
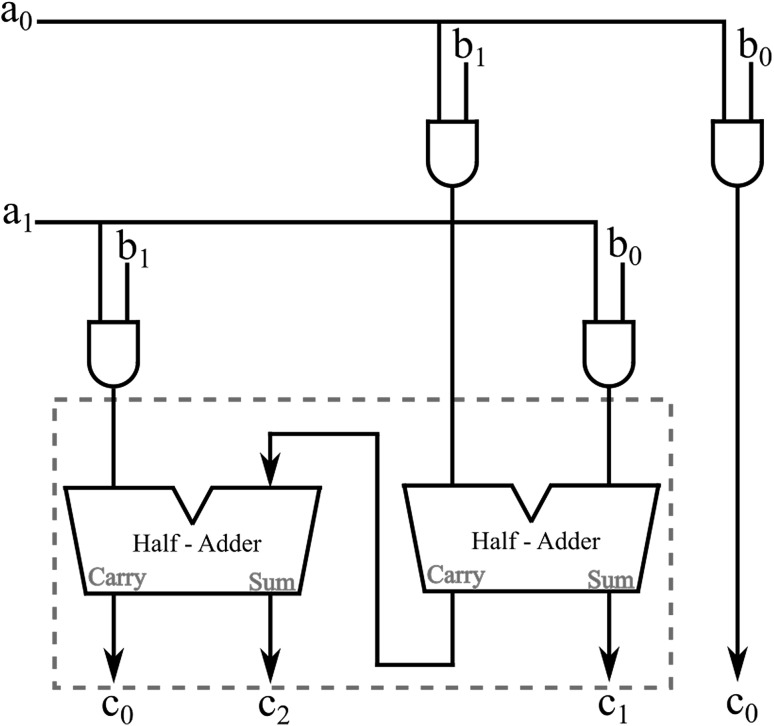
The “Two 2-bit Numbers Multiplier”

In order to present a complete picture of the soliton arrangements as well as the almost unlimited flexibility possessed by the computational system (another reason is that in the view of the author the concept of “Garbage Solitons” is neither entirely satisfactory nor properly defined in its physical terms), in Fig. 18 an alternative soliton arrangement is presented which doesn’t need soliton suppression any more in order for the cascading half-adder arrangement to commence calculation. In this new arrangement the general soliton pattern remains the same as in the original version, with the only difference that now the third control soliton is starting propagation at a time position shifted to the left (top) by four time slots (in Fig. 18 the original third control soliton propagation route has been maintained as well for comparison purposes). The order in which the individual gates are presenting their results is slightly changed as well. Shifting the third control soliton by four time slots to the left (top) of the arrangement has as a result for the soliton carrying the “carry” value to appear at the end (bottom) of the output soliton order. So, this soliton can now be taken as the first input soliton of the new half-adder arrangement (literally, as it possesses the same propagation angle as the original input solitons to the half-adder arrangement) which, by use of a second appropriate input soliton and three control solitons, as required by the scheme, can provide us with the final computational result, without the need to include any kind of soliton suppression procedure.

**Fig. 20:**
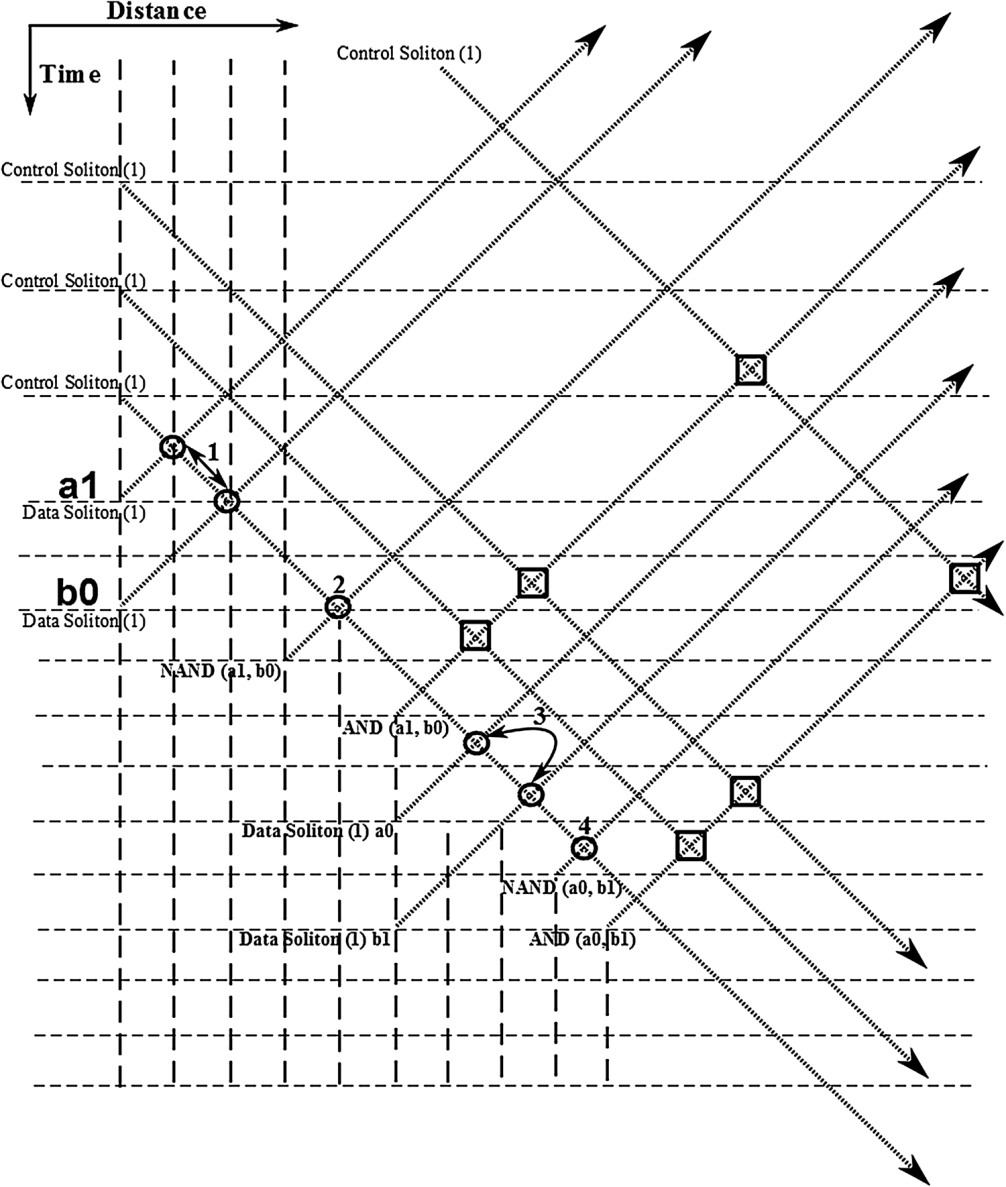
Part of the “Two 2-bit Numbers Multiplier” (including two of the initial AND gates and the half-adder arrangement without the corresponding generated solitons). [Logic gates: (1) NAND, (2) NOT, (3) NAND, (4) NOT]

Having established and demonstrated the three-bit adder arrangement, we can now build around it the full two 2-bit numbers multiplier. The overall arrangement requires the addition of another four AND gates, to accommodate initial bit multiplications. The conventional diagram arrangement for the multiplier is as presented in Fig. 19.

In Fig. 20 part of the two 2-bit numbers multiplier is presented. For illustration purposes generated solitons in Fig. 20 are shown to be closer together than they should be in an actual computational arrangement without loosing in computational properties or upsetting the result. Circular soliton collision points indicate collisions taking place during the initial AND gates calculations, while square soliton collision points indicate collisions taking place as part of the half-adder calculation process. The arrangement in Fig. 20 illustrates a certain degree of parallelism in the calculation process, which contributes significantly in increasing the overall computational speed of the arrangement. It comes without saying that the two 2-bit numbers multiplier arrangement illustrated can be extended to cover any bit length required for the multiplication between two individual numbers. Again, the purpose here was to keep the length of the illustration to a minimum.

## The “butterfly” soliton arrangement

For the remaining part of the “Butterfly” calculation process, we need a soliton arrangement to convert a positive bit-number to a negative one. In order to achieve this we adopt the method of complementing each digit in a bit-number in turn (change 1 for 0 and 0 for 1) and then add 1 to the result. That way, the bit-number taken out of the procedure corresponds to a bit-number representing the negative equivalent of the initial bit-number.

**Fig. 21:**
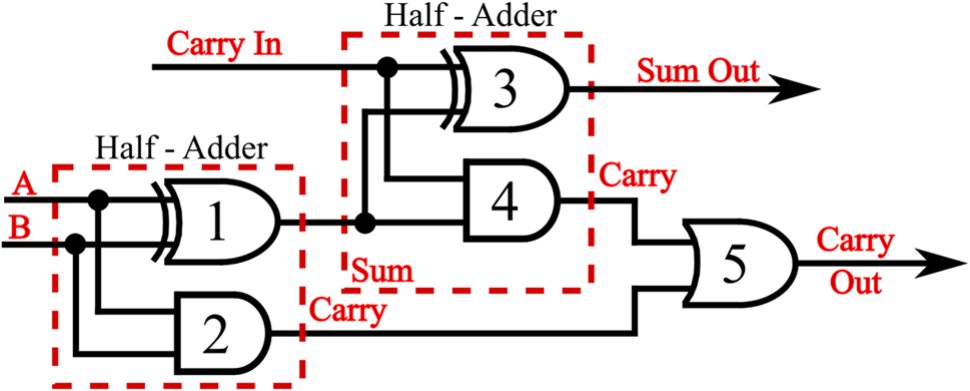
The full-adder (conventional logic arrangement)

A series of collisions between the solitons carrying the bit-number values and a single control soliton with a phase value opposite to the one possessed by the control soliton that generated the initial bit-number, is enough to produce the bit-number complement. Since all the control solitons used so far in the computational arrangements presented had a phase value of $\pi$, corresponding to a bit value of 1, the appropriate control soliton to achieve the complement calculation must possess a phase value of 0, in turn corresponding to a bit value of 0. The addition of 1 to the complement can be easily achieved by means of full-adder arrangements internally consisting of two interconnecting half-adder arrangements and an OR gate, according to the conventional logic scheme presented in Fig. 21.

**Fig. 22:**
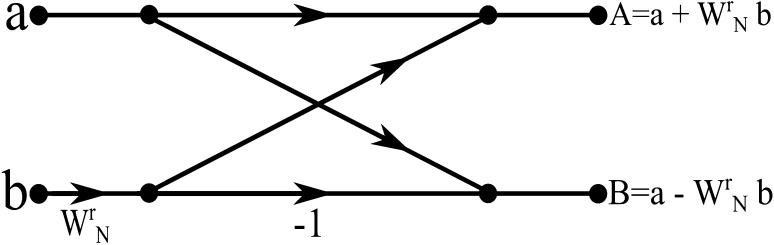
Basic “Butterfly” computation in the decimation-in-time FFT algorithm

**Fig. 23:**
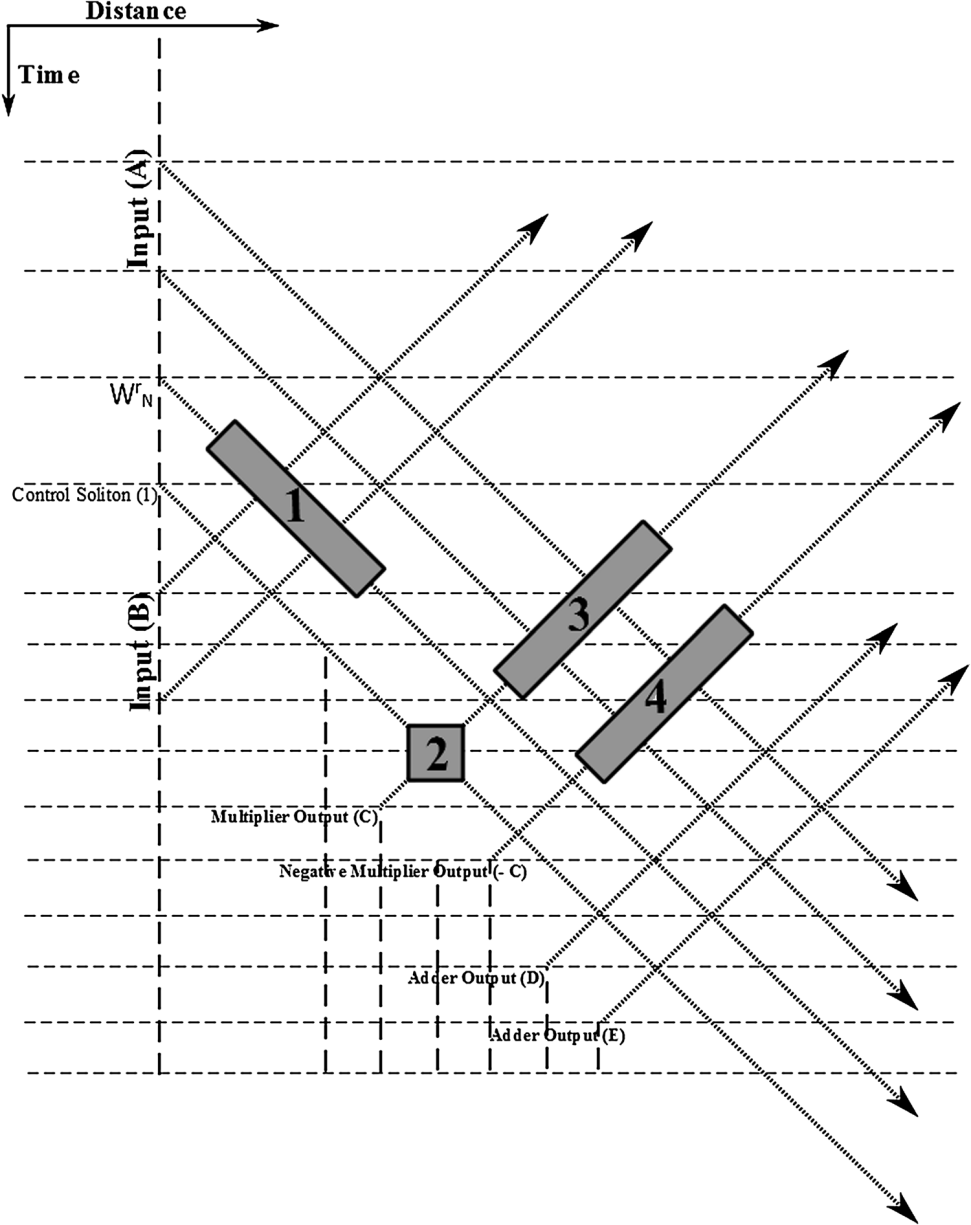
The “Butterfly” soliton arrangement. [(1) Multiplier arrangement, (2) Negation arrangement, (3) Addition arrangement, (4) Addition arrangement]

After the complement of a bit-number has been calculated, subtracting it from another bit-number requires the addition between the complement calculated and the second bit-number. That way only half-adder and full-adder arrangements are required for the realisation of all the calculations involved in the “Butterfly” arrangement. Addition and subtraction calculations appear at the final stages of the “Butterfly” (Fig. 22), those that actually are giving the result and passing the values calculated to the next processing stage of the overall FFT calculation arrangement.

Having completed the presentation of the individual parts out of which the soliton “Butterfly” arrangement consists of, we can now present the schematic of the overall arrangement required. Fig. 23 presents the soliton “Butterfly” arrangement to full extend omitting, by means of a “black box” representation, those parts of the arrangement which have been previously analysed and illustrated. “Adder Output” (D) and “Adder Output” (E) appear at the end of the arrangement as required for the cascading “Butterfly” arrangements to continue further processing the data. All the output soliton propagation routes shown are indicative, since in an actual calculation of bit-numbers more than one solitons will represent the output bit-number of each block of calculation. As it is the case with the conventional Radix-2 FFT algorithm the first and the second decimation process results in a “shuffling” of the input data sequence, which has a well-defined order.

## Conclusions and further research directions

In this paper we surveyed the possibilities of an all-optical soliton FFT calculation and shown how this can become possible within the boundaries of the optical soliton 3NLSE domain. The outcome of this investigation is leading the way towards a fast all-optical soliton FFT calculation with the FFT phasors (roots of unity) to be represented directly by solitons of corresponding phase values. In such a scheme the 8-point FFT phasors, for example, can be directly represented as:

$$\begin{aligned}&{W}_{8}^{0} \rightarrow Soliton \ phase \ value = 2\pi \\&{W}_{8}^{1} \rightarrow Soliton \ phase \ value = \frac{\pi }{4}\\&{W}_{8}^{2} \rightarrow Soliton \ phase \ value = \frac{\pi }{2}\\&{W}_{8}^{3} \rightarrow Soliton \ phase \ value = \frac{3 \pi }{4}\\ \\&{W}_{8}^{4} \rightarrow Soliton \ phase \ value = -2 \pi \\ \\&{W}_{8}^{5} \rightarrow Soliton \ phase \ value = \frac{5 \pi }{4}\\&{W}_{8}^{6} \rightarrow Soliton \ phase \ value = \frac{6 \pi }{4}\\&{W}_{8}^{7} \rightarrow Soliton \ phase \ value = \frac{7 \pi }{4}\\&{W}_{8}^{8} \rightarrow Soliton \ phase \ value = 2 \pi \end{aligned}$$

while the soliton phase values of $\pi$ and 0 remain reserved to represent digit 1 and digit 0 respectively for the control and data solitons involved. This additional ability, when properly specified, will provide the overall computational scheme with a separate, well defined, and of a smaller fixed length FFT calculation arrangement without the need for it to consist of individual calculation arrangements based on the scheme’s “gates”.
